# Planning against Biological Terrorism: Lessons from Outbreak Investigations

**DOI:** 10.3201/eid0905.020388

**Published:** 2003-05

**Authors:** David A. Ashford, Robyn M. Kaiser, Michael E. Bales, Kathleen Shutt, Amee Patrawalla, Andre McShan, Jordan W. Tappero, Bradley A. Perkins, Andrew L. Dannenberg

**Affiliations:** * Centers for Disease Control and Prevention, Atlanta, Georgia, USA

**Keywords:** Bioterrorism, Preparedness, Outbreak, Anthrax, perspective

## Abstract

We examined outbreak investigations conducted around the world from 1988 to 1999 by the Centers for Disease Control and Prevention’s Epidemic Intelligence Service. In 44 (4.0%) of 1,099 investigations, identified causative agents had bioterrorism potential. In six investigations, intentional use of infectious agents was considered. Healthcare providers reported 270 (24.6%) outbreaks and infection control practitioners reported 129 (11.7%); together they reported 399 (36.3%) of the outbreaks. Health departments reported 335 (30.5%) outbreaks. For six outbreaks in which bioterrorism or intentional contamination was possible, reporting was delayed for up to 26 days. We confirmed that the most critical component for bioterrorism outbreak detection and reporting is the frontline healthcare profession and the local health departments. Bioterrorism preparedness should emphasize education and support of this frontline as well as methods to shorten the time between outbreak and reporting.

Bioterrorism is the intentional use of microorganisms or toxins derived from living organisms to cause death or disease in humans, animals, or plants on which we depend.

In 2001, *Bacillus anthracis* was disseminated through the U.S. postal system (*1*). Before that event, concern about bioterrorism had led to preparedness efforts, including strategic planning (*2*). As part of these efforts, we examined investigations conducted by the Centers for Disease Control and Prevention’s (CDC) Epidemic Intelligence Service (EIS). EIS was established after World War II, in part to protect the United States against bioterrorism. We reviewed characteristics and trends of EIS investigations conducted from 1988 to 1999 (*3*). Outbreak investigations from 1946 to 1987 had already been reviewed (*4*). We examined field investigations involving agents that could potentially be used for bioterrorism because understanding how these outbreaks were detected and reported might improve early detection and reporting of bioterrorism.

Each EIS field investigation follows an official request from a state or international health agency. States and international health agencies receive reports of cases or outbreaks from many sources, including local public health agencies, hospitals, healthcare providers, private citizens, or other federal or international agencies (*4*).

We describe lessons learned from outbreak investigations that involved biologic agents with potential for bioterrorism. In addition, we review investigations in which intentional contamination was considered as a potential cause of the outbreak.

## Methods

A standardized form was used to collect data from each investigation from 1988 to 1999. Trip reports submitted by EIS officers after the investigations served as primary sources of information. We focused on outbreaks caused by biologic agents with high potential for bioterrorism, such as *B. anthracis*, *Yersinia pestis*, *Francisella tularensis*, variola virus, viral hemorrhagic fever viruses, *Clostridium botulinum* toxin, *Vibrio cholerensis*, *Rickettsia rickettsiae*, encephalitis viruses, *Brucella* species, *Burkholderia mallei* and *B. pseudomallei*, and others according to our preparedness plans (*2*). We also identified outbreaks in which bioterrorism or intentional contamination was considered. Because each outbreak represented possible bioterrorism, we examined outbreaks in which the etiologic agent remained unidentified. From the tip reports, we abstracted information on possible bioterrorism, causative agent, location, time from first case to first report of the outbreak, and source of recognition and reporting of the outbreak.

We defined the source of recognition and reporting as the person, persons, or institution that originally brought the outbreak or health emergency to the attention of health authorities, as recorded in the trip report. While diagnosis and reporting may be ongoing during an investigation, the initial recognition of an outbreak is a singular event that can occur at the peripheral or primary care setting or at the local, state, or federal level.

We defined the beginning of the outbreak as onset of illness in the first case of the outbreak cluster. Diagnosis of the first illness in an outbreak may occur before the epidemic is recognized and is often determined retrospectively. Epi Info 6 software (CDC, Atlanta, GA) was used to enter the data from the abstractions of the trip reports. SAS software, release 6.12, (SAS Institute Inc., Cary, NC) was used to generate descriptive statistics.

## Results

Several agents have been identified as likely to be used in bioterrorism (2). Of the 1,099 investigated outbreaks, 44 (4.0%) were caused by an agent with potential for bioterrorism (Table 1). *V. cholerensis* was responsible for 18 outbreaks, *Y. pestis* for 11, viral hemorrhagic fever for 7, *Bacillus anthracis* for 3, and *C. botulinum* toxin for 3. *F. tularensis* and *R. rickettsiae* accounted for one outbreak investigation each. The causative agent was not identified in 41 (3.7%) investigations.

**Table 1:** Epidemic Intelligence Service field investigations involving unknown agents and potential agents of bioterrorism, 1988–1999

Agent	Frequency	% of investigations (n = 1,099)
Unknown infectious agent^a^	41	3.7
*Vibrio choleresis*	18	1.6
*Yersinia pestis*	11	1.0
Viral hemorrhagic fever virus	7	0.6
*Bacillus anthracis*	3	0.3
*Clostridium botulinum*	3	0.3
*Coxiella burnetii*	1	0.1
*Francisella tularensis*	1	0.1
Total	85	7.7

^a^In these cases, the outbreak was considered to be caused by an infectious agent because of the characteristics of the illness and outbreak.

The 44 outbreaks involving an agent with potential for bioterrorism and the 41 caused by unknown infectious agents are summarized by location, year, disease agent, and conclusion (Table 2). All botulism outbreaks (two in the United States) were linked to contaminated food. Ten of the 11 plague outbreaks occurred in U.S. areas of known endemic plague in animals. Of the 18 cholera investigations, 4 were in the United States and involved nursing home patients, imported food, raw fish, and contaminated food on an international flight. Twelve (29%) of the 41 outbreaks caused by an unknown agents involved cruise ships.

**Table 2:** Trip reports, involving unknown infectious agents or potential agents of bioterrorism (ultimately not considered bioterrorism), the Centers for Disease Control and Prevention, January 1988–December 1999

Report no.	Y	Location	Disease/agent	Conclusion
90-56	1990	Texas, USA	Unknown	Rash and fever in children, no discernable cause
93-02	1992	Wyoming, USA	*Coxiella burnetii*	Q fever in two bentonite miners
94-02	1993	Georgia, USA	*Clostridium botulinum*	Botulism outbreak linked to contaminated food
94-32	1994	Five states, USA	Unknown	Cluster of cases, no discernable cause
94-42	1994	Texas, USA	*C. botulinum*	Botulism outbreak linked to contaminated food
94-86	1994	Connecticut, USA	Sabia virus	Accidental infection with Sabia virus in laboratory worker
94-88	1994	Bolivia	Machupo virus	Bolivian hemorrhagic fever outbreak
95-16	1994	Utah, USA	Unknown	Contaminated solution used in grafting procedure; source undefined
95-40	1995	Palau	Dengue type 4 virus	Dengue type 4 virus outbreak
95-55	1995	Kikwit, Zaire	Ebola virus	Ebola hemorrhagic fever outbreak
95-61	1995	South Dakota, USA	*Francisella tularensis*	Tick-borne tularemia
98-23	1998	Kenya; Somalia	Rift Valley fever virus	Rift Valley fever outbreak
98-28	1998	Argentina	*C. botulinum* toxin	Botulism outbreak linked to contaminated food
98-35	1998	Uganda	Rift Valley fever virus	Rift Valley fever virus outbreak
98-55	1998	Texas, USA	*Bacillus anthracis*	Exposure to live spore vaccine for anthrax
98-83	1998	Kazakhstan	*B. anthracis*	Reemergence of anthrax, Kazakhstan
11 investigations involving plague	Multiple	10 investigations in USA (Oklahoma, Arizona, New Mexico, Texas, California); one in India.	*Yersinia pestis*	Mostly in areas of endemic plague in animals
18 investigations involving cholera	Multiple	4 investigations in USA (Mississippi, Maryland, Hawaii, California), 14 elsewhere	*Vibrio choleresis*	Cholera in two nursing home patients, outbreak involving imported food, outbreak involving consumption of raw fish, and outbreak involving contaminated food on international flight
12 investigations involving unknown agent on cruise ships	Multiple	Cruise ships	Unknown	Gastroenteritis outbreaks in which infectious agent was not identified by laboratory testing
26 additional investigations involving unknown agent	Multiple	24 in USA, 2 elsewhere	Unknown	Gastroenteritis outbreaks, acute illness after surgical procedures, and other outbreaks in which no infectious agent was identified by laboratory testing

Intentional use of infectious agents to cause harm to civilians (i.e., bioterrorism) was considered in six investigations (Table 3) (*5*–*8*). Although the event did not occur during the period of this review, we included an outbreak of salmonellosis associated with contamination of a salad bar in Oregon in 1984. Several years after the investigation, contamination was (during the study period) determined to be intentional.

**Table 3:** Epidemic Intelligence Service investigations in which bioterrorism or intentional contamination was considered a cause

Report No.	Outbreak	Conclusion
84-093	Salmonellosis, Oregon, 1984	A total of 751 persons became ill with salmonella gastroenteritis. Religious group deliberately contaminated salad bars. *Salmonella* *enterica* Typhimurium strain found in laboratory at commune was indistinguishable from outbreak strain (5).
97-008	*Shigella dysenteriae* type 2, Texas, 1996	Diarrheal illness in hospital laboratory workers who ate pastries, anonymously placed in break room. Identical strains of *S. dysenteriae* type 2 were isolated from stool cultures of case patients, from recovered muffin, and from laboratory stock culture, part of which was missing.
98-006	*S. sonnei*, New Hampshire, 1997	Seven laboratory workers at local hospital became ill with gastroenteritis. Most cases caused by strain of *S. sonnei* that was highly related to a stock culture strain maintained by the hospital laboratory. Possibility that first two cases were caused by intentional contamination could not be excluded.
99-025	Anthrax hoaxes, 1998	Centers for Disease Control and Prevention received reports of alleged anthrax exposure; letters were sent to health clinics in Indiana, Kentucky, and Tennessee and to private business in Tennessee; three telephone threats of anthrax contamination of ventilation systems were made to public and private buildings; all threats were hoaxes.
99-059	Unexplained critical illness, New Hampshire, 1999	A 38-year-old woman was admitted to a hospital with fever, myalgia, and weakness; severe illness and death occurred 32 days after hospital admission; serum specimens indicated *Brucella* species. Patient’s history of multiple febrile illnesses suggested unspecified autoimmune process.
99-94-1	Encephalitis cluster, New York City, 1999	Several residents were hospitalized with illness of unknown etiology characterized by fever, encephalitis, axonal neuropathy, and flaccid paralysis (unpublished data: Epi-1 report); increase in deaths of New York City birds, especially crows; human and bird tissue samples were positive for West Nile-like virus.

Healthcare providers were the source of 270 (24.6%) reports, and infection control practitioners were the source of 129 (11.7%). Together, these two categories of healthcare professionals were the most common source of outbreak recognition and reporting 399 (36.3%). Health departments accounted for 335 (30.5%) reports. Some of these 335 outbreaks may have been originally reported to local health departments by clinicians or clinical laboratories, but the original reporting source may have been missing from the trip report. Other sources of recognition and reporting of these outbreaks were existing surveillance systems (55, 5.0%), foreign ministries of health (30, 2.7%), nongovernmental organizations (22, 2.0%), the World Health Organization (16, 1.5%), and the Indian Health Service (12, 1.1%). Forty-nine (4.5%) outbreaks were reported by other sources, such as private clinics, laboratories, or private citizens. More than one reporting source was found in 58 (5.3%) cases. In 123 (11.2%) outbreaks, no mention was made of the recognition and reporting source, the method of recognition and reporting was unclear, or both the source and the method of recognition and reporting were unclear.

The number of days from the beginning of the outbreak to the date the problem was first identified by the agency requesting CDC assistance was 0 to 26 days (Table 4). The time from the date the initial patient became ill to the date the initial contact was made to the requesting agency for the unexplained critical illness investigation was 26 days (Epi-Aid 99-59). The number of days from the date the problem was identified by the requesting agency to the date of initial CDC contact was 0 to 6 days.

**Table 4:** Number of days from beginning to notification for outbreaks in which bioterrorism or intentional contamination was considered

Report no.	Investigation	Beginning of outbreak	No. of days from first case to problem identification	No. of days from problem identification to initial CDC contact
84-93	Large salmonellosis outbreak caused by intentional contamination of restaurant salad bars, Oregon	9/15/84	6	4
97-008	Shigellosis outbreak in hospital laboratory workers, Texas	10/29/96	1	1
98-006	*Shigella sonnei* outbreak in laboratory workers, New Hampshire	9/20/97	17	3
99-25	Anthrax hoaxes	10/30/98	0	0
99-59	Unexplained critical illness, New Hampshire	3/24/99	26	1
99-094	Encephalitis cluster with paralysis of unknown etiology, New York (West Nile virus)	8/9/99	14	6

^a^CDC, Centers for Disease Control and Prevention.

## Discussion

Investigations from 1988 to 1999 included outbreaks caused by *B. anthracis* spores, *V. cholerae, Y. pestis, F. tularensis*, *Coxiella burnetii*, Venezuelan equine encephalitis virus, viral hemorrhagic fever virus, and *Clostridium botulinum*; all of these agents might pose a bioterrorism threat, were responsible for 4% of all outbreaks from 1988 to 1999, and are not common causes of outbreaks investigated by CDC. A single case of illness or death caused by any of these organisms should suggest intentional exposure (or accidental exposure in which the perpetrators inadvertently exposed themselves to the causative agent.)

However, not all bioterrorism has involved or will involve these high-threat (formerly identified as weaponized) agents. In 1997, a laboratory worker intentionally contaminated his co-workers’ food with a strain of *Shigella* stolen from the laboratory (*9*). While the *Shigella* strain did cause severe gastroenteritis and several hospitalizations, the use of this strain deviates from the popular idea of a bioterrorist’s preferred weapon. However, viewing the bioterrorist’s preferred weapon as a high-threat, aerosolizable, infectious agent that may cause large and fast outbreaks may mislead preparedness efforts. In 1984, the outbreak of salmonellosis associated with intentional contamination of a salad bar in Oregon was not initially considered intentional (*8*); however, further investigation proved that it was. Intentional contamination may resemble naturally occurring outbreaks, may spread slowly through a population, and may involve endemic pathogens. Because of the potential similarity between naturally occurring and intentional outbreaks and the increased threat of bioterrorism in the United States, the index of suspicion for intentional exposure should be high.

Despite advances in the identification of pathogens, outbreaks of unexplained illnesses continue to occur. In this review, we found 41 outbreaks in which the causative agent remained undetermined. Intentional contamination should be considered in these cases because 1) unusual or not easily explained outbreaks are more likely to be caused by intentional contamination, 2) outbreaks resulting from bioengineered pathogens may have unusual or unexpected characteristics, and 3) bioengineered pathogens may not be easily detected by existing assays. For these reasons, outbreaks with unexpected or unusual clinical or epidemiologic characteristics should be pursued with added urgency, and investigators should consider the possibility of previously unidentified or newly engineered pathogens.

While CDC is often notified about outbreak investigations by a state or national health department, the origins of these reports are diverse and include local health departments, surveillance systems, physicians, veterinarians, infection control practitioners, organizations (e.g., the U.S. Food and Drug Administration or the World Health Organization), laboratories, private citizens, ship doctors, vessel sanitation programs, and others. We found that physicians and infection control practitioners reported more than one third of outbreaks. This estimate is probably low because the reports that were recorded as originating from local or state health departments were probably also discovered and first reported by frontline healthcare practitioners (without record of the source). Because of the importance of this frontline in detection and reporting, preparedness efforts must include education and support of these healthcare professionals.

Trip reports (Epi-2) are summaries, not finalized data and are written for the state and local health departments and CDC and the U.S. Department of Health and Human Services. They are primarily internal documents and are not independently peer reviewed or standardized; however, each investigation may use standardized techniques. In general, problems we encountered were not inaccuracies (when a subset of trip reports were compared to the articles that followed them) but rather incompleteness of data we were interested in reviewing. We suggest that trip reports include standardized data collection on certain variables important in evaluating the effectiveness of detecting and reporting outbreaks (e.g., source of outbreak detection, date of the first case diagnosis, and date the outbreak was recognized). Bioterrorism preparedness efforts should include education of frontline public health workers (healthcare practitioners and their supporting clinical laboratory workers). The clinical laboratory should have the capacity and legal latitude to use all appropriate testing. This capacity should include Gram stain of tissue smears for agents such as *B. anthracis*.

Because we cannot rely on astute healthcare practitioners alone, existing national health surveillance systems should be modified or strengthened to increase their effectiveness in identifying bioterrorism (*10*). Surveillance of laboratory data is also a potential (though sometimes delayed) source of identifying changes in disease patterns, and these reporting systems can also be used in bioterrorism surveillance. Improved surveillance for unexplained critical illness and death may also be an important component in improved health surveillance for bioterrorism (*5*).

In addition to healthcare providers and public health departments, other persons and organizations may identify and report outbreaks. For example, veterinarians may be the first to see evidence of bioterrorism because pets and livestock may be more susceptible than humans to agents released in the environment or because susceptible animal population density may be high in the affected area (*11*). An intentional outbreak may mimic a naturally occurring outbreak, such as the West Nile virus outbreak in which the detection of illness in birds and zoo animals was essential for identifying the outbreak. Other potential resources include persons not in the healthcare field. Employers may notice a high rate of illness in their employees, or schools may report a larger than usual absentee rate. Enhancing surveillance systems, providing a mechanism of instant reporting to the proper officials, educating healthcare providers and others in the community, and strengthening knowledge of thorough outbreak investigation will improve collective preparedness for a bioterrorism. In the future, shortening the time from detecting to reporting an outbreak to public health authorities, including CDC, will be essential to an effective response. National health surveillance systems are an important adjunct that, with strengthening, may allow for early detection of bioterrorism. Finally, education about bioterrorism should go beyond the discussion of pathogens such as anthrax and botulism to include the teaching of the critical role and principals of thorough outbreak investigation in protecting the public’s health.
